# Challenges in the Fabrication of Biodegradable and Implantable Optical Fibers for Biomedical Applications

**DOI:** 10.3390/ma14081972

**Published:** 2021-04-15

**Authors:** Agnieszka Gierej, Thomas Geernaert, Sandra Van Vlierberghe, Peter Dubruel, Hugo Thienpont, Francis Berghmans

**Affiliations:** 1 Brussels Photonics (B-PHOT), Department of Applied Physics and Photonics, Vrije Universiteit Brussel and Flanders Make, Pleinlaan 2, B-1050 Brussels, Belgium; thomas.geernaert@vub.be (T.G.); sandra.vanvlierberghe@ugent.be (S.V.V.); hugo.thienpont@vub.be (H.T.); francis.berghmans@vub.be (F.B.); 2 Polymer Chemistry and Biomaterials Group (PBM), Centre of Macromolecular Chemistry (CMaC), Department of Organic and Macromolecular Chemistry, Ghent University, Krijgslaan 281 S4-bis, B-9000 Ghent, Belgium; peter.dubruel@ugent.be

**Keywords:** biomedical materials, optical polymers, polymer optical fibers, fiber fabrication

## Abstract

The limited penetration depth of visible light in biological tissues has encouraged researchers to develop novel implantable light-guiding devices. Optical fibers and waveguides that are made from biocompatible and biodegradable materials offer a straightforward but effective approach to overcome this issue. In the last decade, various optically transparent biomaterials, as well as different fabrication techniques, have been investigated for this purpose, and in view of obtaining fully fledged optical fibers. This article reviews the state-of-the-art in the development of biocompatible and biodegradable optical fibers. Whilst several reviews that focus on the chemical properties of the biomaterials from which these optical waveguides can be made have been published, a systematic review about the actual optical fibers made from these materials and the different fabrication processes is not available yet. This prompted us to investigate the essential properties of these biomaterials, in view of fabricating optical fibers, and in particular to look into the issues related to fabrication techniques, and also to discuss the challenges in the use and operation of these optical fibers. We close our review with a summary and an outline of the applications that may benefit from these novel optical waveguides.

## 1. Introduction

The last decade has witnessed an increasing interest in the development of optical waveguides and fibers made from materials that are biodegradable and biocompatible, in view of serving various medical applications. They can, for example, be implanted during surgery and left inside the human body for the period required to monitor a specific healing process. Once implanted, and after having fulfilled their mission, the bioresorbable waveguides can be left to degrade and be eliminated via natural pathways. Implantable optical waveguides can, not only be implemented to assist the straightforward delivery of light inside the human body, but also to support more advanced tasks, including optogenetic stimulation [1], photodynamic therapy [2], cell imaging [3], and biological sensing [4]. The open literature already reports on the development of biodegradable optical fibers made from a plethora of different materials for potential biomedical applications [5,6,7,8,9]. In this review article, we focus on two main challenges involved when developing such waveguides: the fabrication process, and their eventual performance seen from an optical standpoint. The first challenge relates to the material from which the waveguides are made and how the waveguides are manufactured. The second relates to the duration over which these waveguides can operate, considering their limited lifetime, which is governed by their degradation rate.

**Figure 1:**
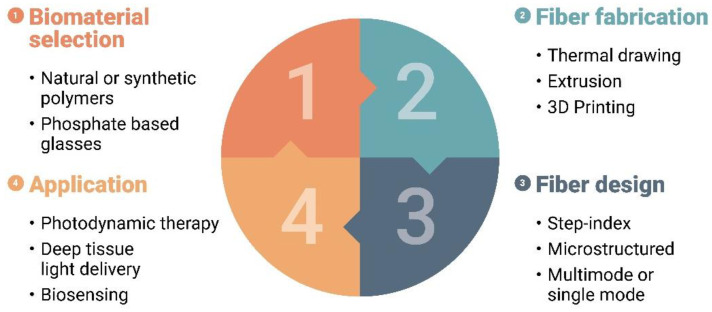
Schematic representation of the four major challenges involved when developing biodegradable and biocompatible optical fibers.

Figure 1 summarizes upfront how the four major aspects of the development of biocompatible and biodegradable optical fibers are interrelated. The selection of the right biomaterial, the actual fiber fabrication technique employed, and the design of the fiber greatly impact the application potential. In this review, we intend to look into these challenges, with a particular emphasis on fabrication approaches, and we will come back to Figure 1 at the end of this manuscript. More specifically, we review different fabrication strategies, as well as the common characteristics of the most employed biomaterials that impact the waveguide fabrication and final performance. We pay particular attention to the methods that allow mass manufacturing of biocompatible optical fibers, in view of ensuring optimized production processes that can deliver products meeting the highest standards in a repeatable manner. We also aim to provide a clear overview of the development of biomaterial-based optical fibers, with an emphasis on their practicality. Finally, we discuss future directions for developing biodegradable and biocompatible optical fibers and hint at potential applications.

The remainder of our paper is structured as follows. In Section 2, we examine the most important materials used for developing implantable optical fibers and focus on a representative group of biomaterials. We highlight their advantages and disadvantages, to provide a clear understanding of the influence of the material on the eventual device performance. In Section 3, we report on the various techniques used for manufacturing biocompatible and biodegradable optical fibers, and by doing so, we summarize the current state-of-the-art in this field. Section 4 provides an overview of the remaining issues and discusses the demands of high quality optical fiber performance upon implantation. We close our manuscript with Section 5, including a summary supplemented with perspectives for future research.

## 2. Material Properties Relevant for Implantable Optical Fibers

From an optical standpoint, the most important features of a material used to fabricate optical waveguides are the optical transmittance and the refractive index. The refractive index of an optical waveguide material must exceed that of the surrounding medium, to support total internal reflection and hence propagation of optical power within the waveguide. The material should also feature both a considerably low absorbance and low scattering, as this is crucial to achieve low attenuation of the optical power as it propagates down the waveguide. The biomaterials used for the fabrication of optical fibers should also feature adequate mechanical properties. Sufficient elasticity and tensile strength (i.e., 70 MPa for poly(methyl methacrylate) (PMMA) [10]) are important; the material should endure fabrication processes such as fiber drawing. To ease processability and to obtain satisfactory fiber properties, polymer materials should exhibit sufficiently high molar mass (preferable in the range of 10^4^–10^5^ g/mol) to prevent filament breakage under draw-down strain, and preferably be amorphous to enhance the mobility of the molecular chains to disentangle and orient in the fiber direction under strain. Additionally, optical fibers should be sufficiently rigid to allow for implantation, but not too stiff or brittle to avoid damaging the surrounding tissue. The thermal properties of the material (e.g., the glass transition temperature, T_g_, or the melting temperature, T_m_) are therefore crucial in view of the waveguide manufacturing process and their actual use and lifetime. The biomaterials should exhibit a T_g_ above the physiological temperature of 37 °C, and preferably even higher (e.g., above 50 °C). Furthermore, implanted fibers need to be compatible with biological tissues and feature a low cytotoxicity. In other words, the fiber must be capable of long-term contact (from several days to months) with living tissues without causing an unacceptable degree of harm to the surrounding tissue and the organism [11]. Additionally, the fiber should preferably be made from materials that can degrade into products that can be excreted by the human body, and hence disappear after use at a rate that is commensurate with the intended application, to prevent any risks associated with surgical retraction.

Standard fused silica glass features excellently low attenuation and chemical inertness. Whilst it is the most popular material for fabricating optical fibers, such glass fibers are not adequate for implantation and long-term in vivo applications due to their mechanical stiffness, fragility, and brittleness, and due to their lack of in vivo resorbability. Indeed, glass shatters when it breaks, and sharp edges and debris could injure surrounding tissue. The remaining debris would not disappear and would require surgical removal. Standard silica-based optical fibers also feature inferior biocompatibility, which is another significant hindrance for many biomedical applications. In this respect, natural and synthetic polymer-based optical materials offer an interesting alternative, as these can be tailored to provide appropriate physical, chemical, and biological properties and functionalities. Polymers can be more readily functionalized with different surface chemistry techniques, for example to provide sensitivity to specific chemical or biological agents. Polymer fibers do not shatter when they break and some of them degrade following hydrolysis that is catalyzed by enzymes.

The technology of polymer optical fibers (or plastic optical fibers), typically abbreviated as POFs, has greatly evolved in the last decades. The most common base material for POFs is poly(methyl methacrylate), or PMMA. The typical operational spectral window for a PMMA POF is around 650 nm, where the optical loss is of the order of 0.15 dB/m [12]. Whilst this is three orders of magnitude higher than the attenuation coefficient of 0.2 dB/km at 1550 nm featured by standard telecommunication grade silica optical fiber, POFs offer other advantages over silica fibers, such as higher fracture toughness, lower Young’s modulus (around 3.2 GPa for PMMA [13]), and higher failure strain. PMMA POFs are typically employed for short-distance optical communication applications or in healthcare systems [14,15]. Besides plain PMMA, other polymers are also frequently used for the fabrication of POFs, such as its deuterated or fluorinated analogs [16]. Other examples include polystyrene (PS), polycarbonate (PC) [17], amorphous fluorinated polymer CYTOP [18], and cyclic olefins such as ZEONEX [19] or TOPAS [20,21]. Extended reviews about POFs, the materials involved in their fabrication, and their applications can be found in [22,23,24,25].

Although the polymer materials mentioned above feature very interesting optical and mechanical properties, none of them combine biocompatibility with biodegradability. In this respect, biodegradable (both synthetic and natural) polymers (derived from renewable resources) have gained increasing attention, not only due to their application possibilities, but also owing to their relatively straightforward processability. Such biodegradable materials have been widely employed, mainly in packaging, agriculture, and medicine [26]. In the medical field, more specifically, biodegradable polymers are used as sutures, implants, scaffolds for tissue engineering, or as controlled drug delivery systems [26,27], and only recently they have been investigated in the form of optical waveguides and fibers.

Some natural proteins (i.e., silk, collagen, fibrin) and polysaccharides (i.e., cellulose agarose, chitosan) are natural biomaterials that demonstrate excellent biocompatibility and biodegradability, and hence they have already been researched for various medical applications, including drug delivery [28] and tissue engineering [29,30,31]. They may provide benefits over synthetic materials since they can more closely match the biophysical properties of tissues within the human body. However, biologically derived materials are generated by nature without strict control over the processes involved, and are limited in terms of the availability of, or access to, the animal or vegetable resources, and hence they may suffer batch-to-batch variation due to the natural variability in macromolecular structures and their molar mass [32]. Natural polymers, especially the protein-based varieties can be immunogenic [33], i.e., they may trigger an unwanted immune response. In addition, natural biopolymers may carry a risk of transmitting infectious diseases when improperly collected, stored, or manufactured [34]. Another significant problem is their decomposition or pyrolytic modification at temperatures below their melting point, which prevents the thermoforming of these materials according to predefined designs at higher temperatures, for example with melt extrusion [35]. Finally, naturally derived materials are typically characterized by poor optical transmittance.

Synthetic biomaterials are often produced from renewable resources and offer much better material designability, as they are man-made products with adjustable physical, mechanical, and chemical properties. They can be created as degradable materials, meaning that they can be resorbed by the human body, and their degradation profile and optical properties can be tuned and optimized to meet the requirements of various optical devices. When one synthetic biomaterial does not satisfy all the necessities, a combination of various types, by means of blending or copolymerization, may allow meeting all the requirements of the intended application. However, synthetic biomaterials also feature disadvantages, because their structure and composition are not the same as those of native tissues, hence their biocompatibility should always be evaluated. The biocompatibility and flexibility of a biomedical device can be realized, for example, using hydrogels as building materials, which are three-dimensional polymer networks that can occur either as synthetic or natural polymers. Natural hydrogels feature excellent biocompatibility and biodegradability. Due to their high water content, hydrogels resemble natural soft tissue more than any other type of polymeric biomaterials and possess biologically recognizable moieties that support cellular activities [36]. Their major limitations relate to an unsatisfactory mechanical strength [37], difficulties in obtaining a repeatable and controllable product from natural resources due to their batch-to-batch variation [38], and the potential to evoke inflammatory responses [36]. For these reasons, natural hydrogels are often combined with synthetic counterparts. Synthetic hydrogels can be engineered to exhibit more reproducible physical and chemical properties, as they can be molecularly tailored with block structures, molecular weights, mechanical strength, and biodegradability [36]. Their material properties are adjustable, they can incorporate chemical functional groups, and they are able to encapsulate drugs or cells [39]. Additionally, bioactive molecules (i.e., RGD peptides or growth factors proteins, GFs) can be incorporated into the synthetic hydrogel network during or after hydrogel formation for fabricating bioactive hydrogels in order to mediate specific cell functions [36]. A key advantage, in terms of processing, is that they are injectable or can be cast, since they are formed from liquid or soluble precursors and can be crosslinked in situ by thermal- or photo-polymerization [40]. On the other hand, their mechanical properties are often inadequate, as they typically have low tensile strength, resulting in brittleness, which may render them unsuitable for some in vivo applications [35,37].

At the other end of the material spectrum, more specifically that of inorganic materials, degradable calcium-phosphate glasses (PGs) [41] have also been considered for fabricating implantable optical fibers. PGs can be tailored by various fractions of calcium oxide (CaO) and magnesium oxide (MgO), in order to adapt their refractive index and degradation rate. The biggest advantage of PGs is their excellent optical transmission in the visible (VIS) and near-infrared (NIR) range of the electromagnetic spectrum. On the other hand, the rigidness and fragility of phosphate glass may provoke an undesirable immune reaction, which limits their biocompatibility.

The families of biomaterials discussed above, and examples thereof, are summarized in Table 1, together with their main advantages and disadvantages.

## 3. Optical Fiber Fabrication Techniques

Typical polymer optical fiber production techniques applied in industry use either a continuous or a discontinuous process flow. In the first type, all process steps run simultaneously, which ensures a high efficiency and production capacity. Continuous manufacturing techniques include continuous extrusion, melt spinning, and photochemical polymerization [42]. Typical discontinuous techniques involve heat-drawing or batch extrusion. These processes consist of at least two steps that should run separately. Therefore, discontinuous techniques typically deliver shorter fiber lengths than those obtained via continuous processes. An important advantage, however, is that these fabrication techniques are not restricted to a specific polymer type, since no in situ polymerization is needed. All the optical fiber manufacturing technologies mentioned above can provide large amounts of optical fibers (tens or hundreds of meters).

Besides the widespread manufacturing strategies including extrusion, co-extrusion, and thermal drawing of optical fibers from preforms, other processing methods have also been tested, such as direct fiber drawing from the melt and fiber casting in a mold following thermal polymerization or photo-polymerization. These fabrication techniques generate only short lengths of fiber (typically a few centimeters) and are chosen due to difficulties in material processing caused by chemical or mechanical properties or limited amounts of available biomaterial.

### 3.1. Thermal Drawing from Preforms

**Figure 2:**
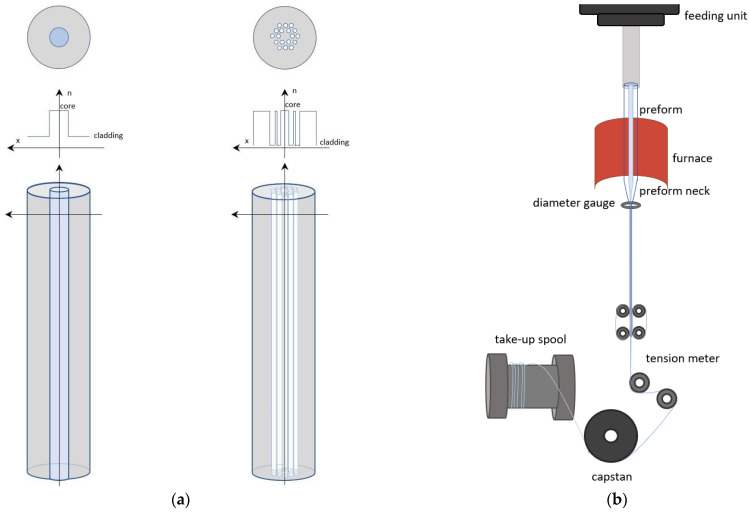
Illustrations of: (**a**) a step-index and a structured preform, with cross- sectional and refractive index profiles; (**b**) principle of the heat-draw process of a preform to make optical fiber.

One of the most widespread fiber manufacturing techniques is the heat-drawing process, which is well known from conventional silica glass fiber manufacturing. It allows delivering kilometers-long optical fibers. Heat-drawing requires two fabrication stages. It starts with a fabrication of a preform from pre-selected materials, which is essentially a scaled-up version of the eventual fiber. A preform already contains a core and cladding structure or an air-hole pattern in a solid rod, to result in, so-called, microstructured or structured optical fibers. The diameter is of the order of centimeters, which is much larger than that of the optical fiber (typically tenths of a millimeter), whilst its length is much shorter. The design of the cross-section of the preform should respect the geometric ratio of the eventual fiber dimensions. The preform can be prepared via several techniques, including: injection molding of molten polymers [43], extrusion [44], casting [45], thin-film rolling [46], stack-and-draw technique [47], 3D printing [48,49], and assembling components via a rod-in-tube technique [50]. The second step of the heat-drawing process involves drawing down this preform into an optical fiber using a heat-draw tower [42]. Illustrations of a preform and a heat-draw process are shown in Figure 2.

Thermal drawing, which is a standardized technique for silica glass fabrication, can also be used for biodegradable and biocompatible fibers. A first, yet peculiar, structure of biodegradable fiber was reported in 2007 [51]. A. Dupuis et al. described a double-core porous fiber structure made from cellulose butyrate (CB) tubes, with lower refractive index polydisperse hydroxypropyl cellulose powder (HPC) suspended in air to obtain an inner cladding. The core consisted of a cellulose tube that could either be collapsed to support light delivery or remain open for potential drug delivery. The fiber was thermally drawn from preforms made from commercially available cellulose butyrate tubes. The preform was first preheated in the furnace of the drawing tower at a temperature of 150 °C for one hour, and then the optical fiber was drawn at about 180 °C to a diameter of around 450 µm. Despite the relatively high attenuation of around 1.1 dB/cm at 630 nm, the fiber showed potential to detect changes in transmission when its pores were filled with deionized water. This publication led to the proposal of a multifunctional fiber that could possibly embody microfluidic functionalities and drug release. A. Dupuis et al. also reported on biodegradable fibers created from biomaterials such as poly(L-lactic acid), poly-ε-caprolactone (PCL), and cellulose derivatives [52]. Hollow core, step-index (SI), and multilayer fibers were thermally drawn from preforms using standard drawing towers. The preform fabrication process included various techniques, such as co-rolling of plastic films to manufacture multilayered preforms consisting of several materials: poly-ε-caprolactone (PCL) powder-filling with a higher refractive index into lower refractive index cellulose butyrate (CB) tubes to form single core and multiple core step-index structures; and solution-casting of hydroxypropyl cellulose inside the CB tubes, which were subsequently solidified in a vacuum oven. All the manufactured fibers featured relatively high attenuation, significantly above 1 dB/cm at 633 nm [52,53]. The authors did not discuss the actual degradation of their implantable optical fibers, nor did they report an evaluation of their biocompatibility.

The use of commercial semi-crystalline polyesters, PLLA and PLGA, in the form of fibers was first described by R. Fu et al. [54]. The authors studied in vivo deep-brain neural activity by means of intracranial light delivery and detection based on fluorescence sensing in living mice. Unclad poly(L-lactic acid) (PLLA) and poly(L-lactic-co-glycolic acid) PLGA optical fibers were fabricated with a simplified thermal drawing process from molten semi-crystalline polyester beads at 220 °C and using glass capillary tubes, resulting in short fiber lengths of 5 cm and a diameter of about 220 µm. The propagation losses of these fibers were also rather high, 1.64 dB/cm at 473 nm in air. The authors also reported the evaluation of the biocompatibility and degradability of the implanted fibers in living mice models. They demonstrated that PLLA-based optical fibers are great candidates for deep tissue light delivery and detection.

In our own work, we have used an amorphous form of PLA, more precisely poly(D,L-lactic acid) (PDLLA), to fabricate optical fibers [55]. PDLLA may be better suited for optical fiber fabrication since, in contrast to PLLA, it is an amorphous material, which indicates that a lower optical loss could be achieved. The manufacturing exploited a standard heat drawing process from previously fabricated preforms in the shape of cylindrical rods, which had been pre-manufactured by melting PDLLA granulates. The unclad PDLLA fiber demonstrated an attenuation coefficient of 0.11 dB/cm at 772 nm, which is the lowest loss reported so far for biodegradable optical polymer fiber. The degradation of the unclad PDLLA fiber followed the trend of the bulk material degradation, which indicated that fibers with the largest diameter of 600 µm degraded faster than those with smaller diameters of 300 and 200 µm and featured more than 84% molar mass loss over a period of 3 months. We also evaluated the optical loss at 633 nm of these unclad PDLLA fibers during immersion in physiological fluid, phosphate-buffered saline (PBS) at 37 °C and pH = 7.4, confirming that PDLLA-based fibers can efficiently deliver light over a period of 30 min, which is commensurate with that required for photodynamic therapy (PDT). In subsequent work, we also reported on the fabrication of SI polyester-based fibers, in which the core consisted of poly(D,L-lactic-co-glycolic acid) (PDLLA) and the cladding of poly(D,L-lactic acid) (PDLLA) [56]. The preforms were prepared by means of a rod-in-tube technique by melting granulates, and the core-cladding fibers were manufactured with a standard heat drawing process. Cutback measurements returned a slightly higher attenuation coefficient of 0.26 dB/cm at 950 nm for fibers with an outer diameter of 1000 ± 50 µm, a core of 570 ± 30 µm, and a numerical aperture of 0.163. These SI PDLGA-PDLLA fibers also demonstrated no additional optical loss caused by immersion in PBS during the first 30–40 min, which made them suitable candidates for PDT.

Very recently, S. Shadman et al. [57] developed specialty optical fibers consisting of poly(D,L-lactic-co-glycolic acid) (PDLGA) combined with PMMA and poly-ε-caprolactone (PCL). The biocompatible fibers were also fabricated by thermal drawing from preforms, and the obtained fibers were available in various shapes, such as rectangular, cylindrical, core-shell, and multi-material planar waveguides with channels placed on the sides of the structure, to permit incorporation within biodegradable polymers. The authors reported meters-long microstructured fibers that showed sufficient flexibility for knotting and weaving. The authors emphasized the possibilities of complex release mechanism profiles via morphological evolution of the degrading polymers in diffusion control delivery. The optical characteristics of these microstructured fibers, however, were not reported.

S. Farajikhah et al. [58] demonstrated the fabrication of PCL based optical fiber with tailored cross-sections, such as unclad solid-core filaments, hollow-core, and grooved fibers, all thermally drawn from preforms. The PCL preforms were prepared by melting PCL pellets at 80 °C for 17 h inside polypropylene and Teflon^®^ molds of various shapes. Subsequently, the PCL based fibers were drawn using a standard heat-draw tower at 85 °C. The optical loss of a PCL hollow core fiber with a 200 μm internal diameter that was sealed prior to immersion in PBS was around 1.5 dB/cm at 635 nm at the beginning of the study (day 0) and increased to around 2.5 dB/cm at 635 nm over 21 days of immersion, which indicates the very stable optical working lifetime for these PCL based fibers. PCL fibers with unclad solid cores, and those with various topographies treated with 0.1% (wt/vol) gelatin, demonstrated good adhesion and proliferation of MCF-7 cells, which suggested that these PCL fibers support cell attachment and growth. Moreover, cells were found to attach to the PCL fibers to a lesser extent also without any prior gelatinization. PCL fibers were demonstrated to be non-cytotoxic and hence suitable for biomedical application, including tissue engineering.

As indicated earlier, resorbable phosphate glasses (PGs) have also been exploited to produce optical fibers intended for use in the biomedical field [41,59]. By adapting the ratios of calcium oxide (CaO) and magnesium oxide (MgO), the refractive index of the PGs was tailored to form a core-cladding structure with a step-index profile. Thermally drawn single-mode fibers had an outer diameter of 120 µm and a core diameter of 12 µm and featured an attenuation as low as 1.86 dB/m at 1300 nm and 4.67 dB/m at 633 nm, which is the broadest wavelength range showing a relatively low propagation loss among all biodegradable optical fibers. This resulted from the fact that glasses are typically highly transparent and that glass fibers benefit from a well-developed manufacturing platform. However, the preform processing of PGs is not standard for typical glass preforms and cannot yet be realized by the conventional modified chemical vapor deposition (MCVD) [60] that is typically employed with silica preforms. Instead, PGs processing allows for preform fabrication by means of a rod-in-tube technique. D. Gallichi-Nottiani and D. Pugliese et al. [61] recently demonstrated the fabrication of microstructured fiber preforms of bioresorbable phosphate glass and subsequent fiber drawing. To form this complex preform, the authors first obtained the outer tube by direct extrusion and subsequently they used a standard stack-and-draw technique [47], for which the preform was prepared by stacking the extruded capillaries within a tube. The authors demonstrated light guidance at 1300 nm, but a full optical characterization was not reported.

Table 2 summarizes biodegradable optical fibers and waveguides fabricated by thermal drawing of a preform as reviewed above.

### 3.2. Extrusion and Extrusion-Based 3D Printing

**Figure 3:**
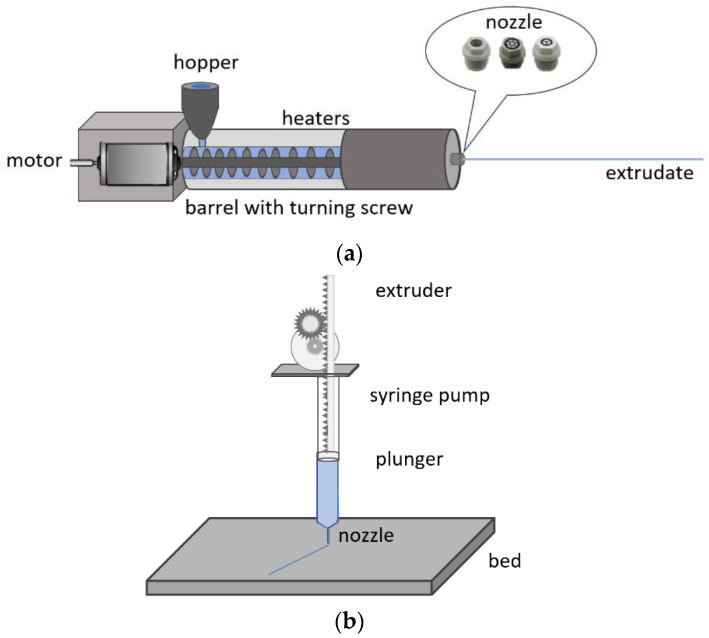
Illustrations of (**a**) optical fiber extrusion, (**b**) 3D extrusion-based printing.

In an extrusion process, a molten material (typically a polymer or a soft glass) is forced through a die with a dedicated pattern. In the case of continuous extrusion, a mixture of monomers, initiators, and additives is extruded in a continuous fashion during which the polymerization reaction occurs inside the reactor while the material is being extruded. Batch extrusion is a discontinuous process and permits a direct polymer fiber fabrication technique, which is applied in two steps. In the first step, monomer, initiator, and other additives, such as dopants, are inserted using a vacuum pump into a reactor, in which the polymerization takes place [42]. After full conversion of these starting materials, the temperature of the reactor is raised to generate a polymer melt. In the second step of the process, the polymer melt is pumped through a spinning nozzle to be extruded in the form of filament or unclad fiber. The batch extrusion can also be carried out starting with commercial polymer pellets or granulates [62]. Extrusion-based additive manufacturing (AM), or so-called 3D printing systems, have already been widely used for fabricating tissue engineering scaffolds [63,64]. The extrusion-based modules typically use high forces and temperatures to dispense materials through a micro-nozzle. Typically, the type of the extruded fiber or preform shape depends on the employed extrusion die. Hence, the extrusion method also allows manufacturing air-holed structures in polymer materials if the employed die has a holey pattern [65], which in its turn enables fabricating microstructured or structured optical fibers. Step-index fiber can also be fabricated using this technique, as different refractive index materials can be co-extruded to obtain core and cladding layers simultaneously [66]. Figure 3 illustrates optical fiber extrusion and extrusion-based 3D printing.

Using this technique, naturally derived spider silk has been employed to form optical waveguides. The extruded silk waveguides were generated through direct ink writing from an aqueous silk fibroin solution by S. Parker et al. [67]. Silk waveguides were printed on borosilicate glass slides in both straight and wavy configurations, with lengths of several centimeters and a diameter of around 5 μm; but they were not handled away from the glass substrate. Their attenuation was around 0.25 dB/cm at 633 nm. Silk fibers were also obtained by direct reeling of fiber collected from the female spider’s major ampullate glands onto a spool under controlled conditions at a constant speed of 5 mm/s. These silk fibers also had a small diameter of approximately 5 μm, which complicates the coupling of light to the fiber. In addition, scattering and absorption of the silk material resulted in large propagation losses, of approximately 10 dB/cm at 630 nm [68].

Recently, PEG-based hydrogel precursors, specifically pre-polymers of poly(ethylene glycol) diacrylate (PEGDA) reacted with thiol groups of DTT (DL-dithiothreitol), have also been investigated for manufacturing optical waveguides [69]. Feng J. et al., worked with an extrusion-based printing technique following in situ photopolymerization using a 3D-bioscaffolder to fabricate hydrogel-based optical waveguides in the form of unclad PEGDA-DTT and step-index fibers, in which PEGDA-DTT formed the core and acrylated Pluronic F127-DA formed the cladding. The SI fibers were printed using a coaxial printing needle with varying core diameters, ranging from 340 to 640 µm and with a fixed outside diameter of 1.02 mm. The authors reported that continuous lengths of 50 cm could be achieved using this fabrication technique. These hydrogel-based fibers showed improved light guidance, featuring optical losses of 0.1 dB/cm at 520 nm, 0.4 dB/cm at 405 nm in air, and 0.25–0.7 dB/cm once inserted in tissue. Printed PEGDA-DTT waveguides demonstrated the possibility of activating optogenetic switches in cells using light delivery, and to control cell adhesion and their migration in a photo-responsive 3D culture of a fibroblast spheroid within a polymeric matrix.

An alternative extrusion method used to fabricate cellulose-based optical fibers included a wet-jet spinning device equipped with a spinning nozzle. H. Orelma et al. [70] reported the fabrication of a core filament that was made from cellulose dissolved in [EMIM] AcO, which was passed through a nozzle into a water coagulation bath that was kept at a constant spinning rate of 0.5 mL/min. After spinning, the filaments were stored in water for 2 h and subsequently dried under tension at room temperature and ambient humidity. The cladding was applied by coating the core filament by the cladding layer using a lab coater. The cladding consisted of cellulose acetate dissolved in acetone that was produced by coating the prefabricated regenerated cellulose filament core with a cellulase acetate. The acetone was subsequently evaporated from the coated filament at room atmosphere overnight. The diameter of the cellulose-based fiber was approximately 210 µm, and the cellulose acetate cladding thickness was 3.40 ± 0.20 µm. The longest fabricated fiber was around 76 mm, and the minimum attenuation of 5.9 dB/cm was found at 1130 nm. In the wavelength range of 750–1350 nm, the optical loss was below 10 dB/cm, which is still considered relatively high for a step-index fiber. The authors indicated the sensing potential of these hydrogel-based fibers by immersing them in water and measuring the increase of the attenuation, which appeared to be a reversible process.

### 3.3. Casting in a Mold and Curing

**Figure 4:**
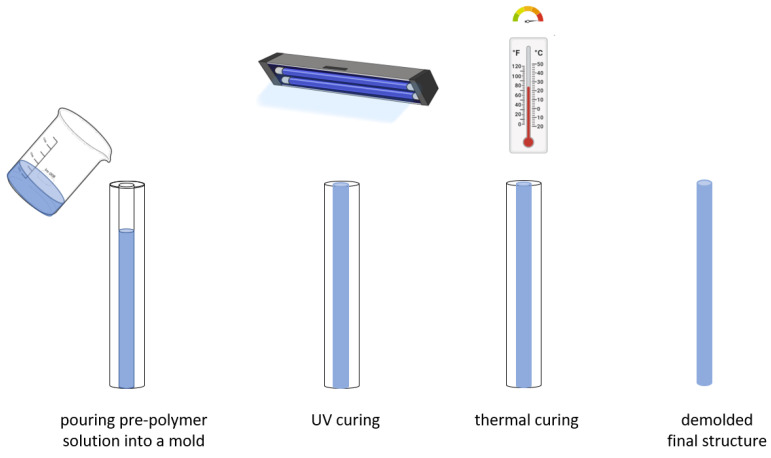
Principle of optical fiber fabrication by casting: pouring pre-polymer solution into a mold followed by either UV curing or thermal curing.

Casting has been typically applied to produce both silica glass and polymer preforms, which were consequently thermally drawn to optical fibers. For glass materials, casting involves low temperature sol-gel technology, in which an intermediate preform is formed by pouring the sol into a mold where it is turned into a gel by lowering its pH. At the wet gel stage, the cast mandrel elements are removed, leaving air columns within the gel body. The gel body is then treated to remove water, as well as organic and transition metal contaminants. The dried porous gel body is subsequently sintered at around 1600 °C into viscous glass and finally drawn into an optical fiber [71]. For polymer materials, the polymer preform is typically formed by in situ chemical polymerization [72]. First, all the necessary chemical precursors (i.e., monomer, initiator, and chain-transfer agent) are inserted into a mold that mirrors the preform geometry. The polymerizing mixture requires degassing to prevent the formation of bubbles, or the reaction should be initiated in vacuum conditions. Another method involves the solution casting of the initial polymer in a suitable solvent (e.g., chloroform), which is followed by drying at room temperature or in vacuum. Alternatively, with degradable thermosets after solution casting, the pre-polymer is cured as it undergoes cross-linking reactions to form three-dimensional networks. The cross-linking reaction happens with the aid of cross-linking agents combined with exposure to high temperature or UV radiation, depending on the material. Once the curing is completed, the solid structure is released from the mold. Figure 4 illustrates a typical casting process followed by curing. The main advantages of casting methods are simplicity, low production cost, and the possibility of machining the mold to obtain preforms or short fibers with arbitrary shapes.

As previously indicated, casting represents a very straightforward method for fabricating short lengths of fibers directly in a mold. Besides this, casting has been used to apply a cladding layer to a previously fabricated unclad optical fiber. This technique was employed to fabricate step-index silk waveguides with a core made of high-index silk fibroin and a hydrogel lower index cladding. The step-index silk fibers were prepared in two steps. First, the silk solution was cast into a mold and after drying into films, the silk films were surrounded by a hydrogel solution cast inside a Teflon^®^ tube prior to gelation. The attenuation coefficient of around 2 dB/cm at 540 nm was mainly caused by the roughness of the silk film [73]. Qiao, X. et al. [74] reported the fabrication of implantable spider silk-based optical waveguides, made both from recombinant spider silk protein and regenerative silkworm silk protein, which was cast into Teflon^®^ tubes with inner diameters of 800 µm. The regenerative silkworm silk featured a refractive index n = 1.52, whilst for the recombinant spider silk this was n = 1.70 at 635 nm. This led to propagation losses of approximately 0.8 dB/cm at 635 nm in air and 1.9 ± 0.3 dB/cm in vivo in mice [74].

M. Choi et al. [75] manufactured a hydrogel-based step-index optical fiber with a two-step process that consisted of a poly(ethylene glycol) (PEG) core and an alginate cladding. First, the core was formed inside a silicone mold in a tube shape. The precursor solution for the PEG hydrogel with a radical photo-initiator (2-Hydroxy-2-methylpropiophenone) was injected into this tube and subsequently photo-crosslinked by exposure to UV light. To retrieve the core from the mold, the mold was immersed in dichloromethane until the silicone mold became swollen and the PEG core could be extracted. Next, the core with a diameter of around 250–800 µm was dipped multiple times in a sodium alginate (SA) solution and calcium chloride solution to obtain physical crosslinking between the carboxyl group (–COOH) of SA to the calcium ion (Ca^2+^) of the CaCl_2_, and by doing so to generate alginate cladding with a desired thickness (typically around 100–150 µm). The authors did not report the total length of the fabricated fibers using this technique, but showed a photograph of a 1 m-long fiber. The fibers demonstrated losses below 0.42 dB/cm across the VIS range, and insertion into the intestine of living mice confirmed that light delivery through tissue was achieved, indicating the potential for deep tissue photothermal or photodynamic therapy. The PEG based core allowed incorporating various functional materials, including organic dyes-(rhodamine 6G, as well as biotin-conjugated fluorophores) for the generation of fluorescence. PEG was also examined by S. Nizamoglu et al. [76], who reported on the manufacturing of planar waveguides from hydrogel for photochemical tissue bonding (PTB) in porcine skin wounds. PEG hydrogel-based planar waveguides were prepared by photopolymerization of a precursor solution containing 80 wt% polyethylene glycol diacrylate, 15 wt% water, and 5 wt% 2-hydroxy-2-methylpropiophenone using a UV lamp. The optical properties of these waveguides were not reported. A very similar approach of solution casting of liquid alginate-polyacrylamide precursor hydrogels was used by J. Guo et al. [77] to obtain hydrogel-based core-cladding optical fibers with an enhanced toughness and stretchability. This was done by means of a hybrid polymer network that contained both ionic and covalent bonds included in hydrogels to upgrade their robustness. The core was fabricated by injecting a solution containing Ca^2+^ alginate polyacrylamide (PAAm) precursor solution into a silicone tube mold with a syringe and UV curing. After polymerization, the core was extracted from the mold by swelling the tube in dichloromethane. Subsequently, the cladding was added directly onto the core by dip-coating and photopolymerized under UV light to form a step-index structure. To ensure robust bonding between the core and cladding, alginate was chemically anchored using EDC/NHS chemistry and additionally, ionic cross-linking of Ca^2+^ in alginate cladding was applied. The fibers were developed for strain sensing and could be stretched repetitively to an axial strain up to 730% without deformation. The authors claimed that these hydrogel-based stretchable optical fibers are suitable candidates for optical strain sensors in wearable devices.

A first step-index biodegradable optical fiber was produced from polyester-based elastomers by D. Shan et al. [78]. In this fiber, the core was made of poly(octamethylene maleate citrate) (POMC), while the cladding consisted of poly(octamethylene citrate) (POC). The step-index optical fibers were manufactured by thermal crosslinking (at 70 °C for 4 days) of a pre-polymer cladding material in the form of a tube surrounding a stainless-steel wire with a diameter 500 µm. Next, to remove the POC cladding tube from the wire, the polymer-coated wire was immersed in 30% ethanol solution overnight, and the POC tube was retrieved from the metal wire after swelling in ethanol. In the third step, the authors used air pressure infiltration of the liquid POMC into the pre-polymerized POC tube, which was followed by thermal crosslinking of both combined parts for another 3 days at 80 °C. D. Shan et al. did not reveal the total fiber length, but they reported on experiments with a longest tested fiber sample of 7 cm. The propagation loss of 0.4 dB/cm at 633 nm allowed delivering enough light deep into tissue, which was tested by placing a fiber under a slice of porcine tissue with a thickness of ~2 mm under bending angles of 0°, 30°, and 90°. Additionally, the authors demonstrated fluorescence sensing in vivo using an agar gel doped with Rhodamine B dye in the abdomen area of a rat. Two fibers with a length of 7 cm were inserted into the animal, first for the dye-excitation at 532 nm, and the second for the collection of the emitted red fluorescent light. D. Shan et al. also showed an early proof-of-concept of image transmission through this citrate-based polymeric optical fiber. They applied spatial patterns on a digital micromirror device projected onto the proximal end of the fiber by a He-Ne laser through an imaging telescope unit. To monitor the pattern projection, a pellicle beam splitter with a splitting ratio of 8:92 (reflection: transmission), in conjunction with an imaging setup, was used. The corresponding output image pattern at the distal end of the citrate-based fiber was registered with a charge-coupled device (CCD) camera. Due to the multimodal light propagation in the fiber, the output projection contained random speckle patterns and did not completely match the input pattern, hence image reconstruction was applied.

Recently, E. Fujiwara et al. [79] reported on the manufacturing of structured agarose optical fibers with a solid core surrounded by six air holes. The fiber was made by directly pouring solubilized agarose into a glass mold (inner diameter = 3 mm) with six internal rods (diameter = 0.5 mm). The agarose-based fiber demonstrated a loss of 3.23 dB/cm at 633 nm, with the prospect of application for in vivo biochemical sensing, as demonstrated by measuring the output speckle intensity as an effect of different fluids inserted into the air holes.

Table 3 summarizes the fabrication techniques for different optical fibers made from a variety of biomaterials, together with their optical loss.

## 4. Challenges in Biocompatible and Biodegradable Optical Fiber Fabrication and Operation

As already highlighted above, the fibers that are the subject of this review must simultaneously meet optical (tailored refractive index, low optical loss), mechanical (adequate mechanical flexibility for tissue compliance), and biological (biocompatibility and adaptable biodegradability) requirements.

**Figure 5:**
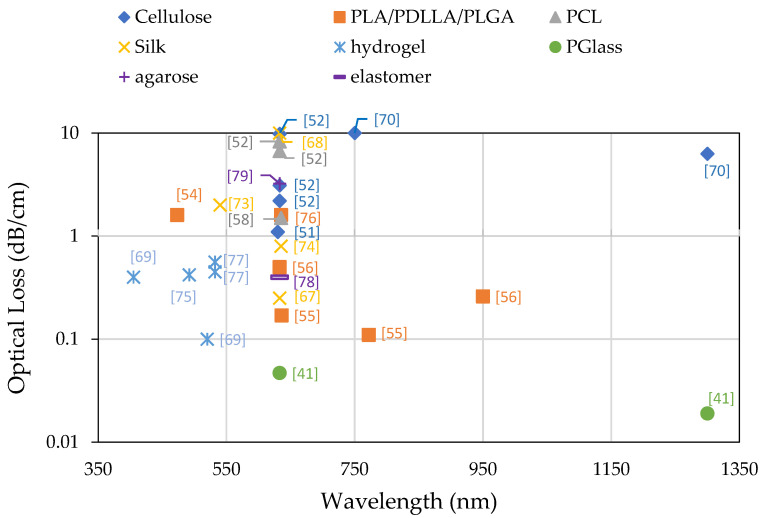
Reported attenuation coefficient of optical fibers made from different biomaterials. The numbers between square brackets indicate the corresponding reference.

One of the most important parameters for an optical fiber is its optical transparency, defined by the attenuation coefficient that reveals the optical loss following propagation over a certain distance down the fiber. The fibers that we have reviewed so far display attenuation coefficients between 10 dB/cm and 0.02 dB/cm, depending on the biomaterial from which they are made, their structure, and the fabrication process, as well as on the wavelength at which the optical loss measurement was conducted. Figure 5 shows the attenuation coefficients for the various fibers as a function of wavelength.

Synthetic polymers (e.g., PLA, PDLLA, and PLGA) seem to provide fibers with superior characteristics over natural polymers, since their properties, e.g., mechanical and physical properties or degradation rate, can be adapted to particular applications. These polymeric biomaterials are commercially available and feature sufficient mechanical strength in view of material processing, as well as maintaining stable working performance. Furthermore, these biopolymers have been the subject of years of scientific research, resulting in several PLA and PLGA-based medical products that are approved and regulated by the US Food and Drug Administration (FDA). In addition, clinical studies of the degradation and biocompatibility of PDLLA and PDLGA have already been reported with no clinical signs of foreign-body reactions to these materials [80]. Significant progress on implantable fibers has also been made with phosphate glass. Such PG reveals excellent transparency, featuring an attenuation coefficient that is one order of magnitude lower than the attenuation of polymer optical fibers [41]. Phosphate based glass fibers proved to be resorbable in vivo [81] and demonstrated good biocompatibility with tissues [81,82]. PG-based fibers demonstrated the potential to be fabricated with a small diameter, yielding single mode guidance. They also allow for microstructuring [61] and for the inscription of fiber Bragg gratings (FBG) [59], which are well-known fiber-based sensor elements that allow projecting advanced biosensing applications. One still needs to account for the fragility of PG-based fibers. They are prone to crystallization, as their viscosities are much lower than silicates [83], hence the fiber drawing might be troublesome and can require rapid quenching or drawing of the fiber directly from a melt [84]. To avoid brittleness, an additional coating could be applied to reinforce PG-based fibers using other polymeric biomaterials.

The selection of the biomaterial primarily impacts the possible approaches for fiber manufacturing. As discussed earlier, three main techniques have been employed for fabricating biocompatible and biodegradable optical fibers, i.e., thermal drawing, extrusion-based printing, and casting. Only the first two enable producing relatively larger fiber lengths. One can therefore conjecture that those have the best potential to deliver a product with a stable batch-to batch consistency, at a competitive cost. Although there is no demand for implanting a fiber that is several meters long in a human body, it is important to deliver excellent products characterized by a repeatable performance.

A third major influence on fiber performance stems from its structural design. Planar optical waveguides with limited capacity to confine and guide or unclad optical fibers are prone to large optical losses as soon as they are embedded into tissue. Step-index or microstructured fibers are likely needed to ensure stable operation in vivo. In addition, the degradation rate of the biomaterial-based fiber governs how long it takes for an implanted fiber to be resorbed, and this largely depends on the biomaterial itself, as well as on the fiber dimensions and on the implantation site. The required operational period of the fiber can vary from minutes to hours, depending on the application scenario. In the case of long-term health monitoring and drug delivery, this can even be days or weeks. So far, the open literature offers very limited data regarding optical losses caused by implantation in vivo or in a simulated biological environment.

At this stage we would like to go back to Figure 1, with which we started our paper, and which attempts to illustrate the discussion above and highlight how the different aspects dealt with so far are interrelated. The selection of a certain biomaterial not only strongly impacts the possible manufacturing technology and the fiber design, but it also affects the application potential. Consider for example a PDT scenario which exploits a photosensitive cancer killing agent that is activated by a specific wavelength emitted by a surgical laser that needs delivery through an implanted optical fiber. One must take into account a biomaterial that shows low loss at that particular wavelength. Additionally, it requires an optical source that provides for a broad and uniform illumination to obtain the most effective and consistent PDT treatment [85], and therefore the fiber should possess a large numerical aperture or its distal end may have to be terminated with a radial diffuser, which could be in the form of cylindrical elongated sections that scatter light sideways to the surrendered diseased tissue. If the treatment requires covering an even larger volume of diseased tissue, the use of several smaller fibers in a bundle should also be possible [85]. This example shows that many simultaneous requirements must be met to ensure an effective use of biodegradable and biocompatible fibers.

## 5. Conclusions and Outlook

We reviewed the state-of-the-art of optical fibers characterized by biodegradability and biocompatibility, confirmed by either in vitro or in vivo studies. We discussed the challenges posed by their manufacturing process depending on the material type. We have seen that, besides conventional thermal drawing and extrusion processes, molding and casting have also been investigated for producing short fibers, starting from several novel biomaterials. Step-index fibers have been achieved by combining these techniques with dip-coating in a lower refractive index hydrogel solution or UV-cross-linkable polymer. This approach leads to very short fibers, which is sufficient for research into the properties of novel biomaterial-based waveguides, but is not adequate for volume production and for delivering meters of fibers with consistent characteristics. We therefore foresee that standard heat-drawing or extrusion are still the most promising production technologies that allow for economically large-scale optical fiber fabrication and that provide sufficient design flexibility, to obtain both step-index and microstructured fibers. Thermal drawing requires preform fabrication, which can be a troublesome task, but the heat-drawn fibers are typically characterized with lower roughness than their extruded counterparts, and hence feature lower optical loss. Extrusion techniques, on the other hand, simplify fiber fabrication as they involve a single continuous process, starting with polymer pellets and ending with the finished fiber. The extruded fiber fabrication process is also possible on a large scale, with limited batch-to-batch variations. The main thrust for both technologies should come from improvements in the fabrication of low-loss standard step-index single-mode or multimode fibers from the most promising optical biomaterials, but also from the development of more complex optical structures, extending beyond conventional core-cladding fibers.

The materials that we have reviewed so far typically feature a tunable degradation rate, biocompatibility, and various mechanical and optical properties. The optical properties remain a key aspect, irrespective of the targeted application. In general the optical performance and processing capabilities of polyesters and other thermoplastics are superior to hydrogel materials, and would therefore be preferred. However, to mitigate against potential tissue damage upon in vivo insertion of a fiber, the application of a hydrogel coating onto a thermoplastic polymer fiber may be a promising strategy to “soften” the surface of such polymer optical fibers.

To the best of our knowledge, there are no biomaterial-based optical fibers that have already been validated for use in a clinical study. Nevertheless, many of the biomaterials discussed in this review have been applied in other medical tools that did receive clinical approval. For those tools, the main requirement for the biomaterial, besides its biocompatibility, is its capability to match the required mechanical properties and its adaptable degradation time given a specific application. To give a few examples, silk fibroin (SF), an FDA-approved natural protein, is commonly used in sutures, surgical meshes, and fabrics for skin wound healing and for tissue engineering by means of scaffolds [86]. Agarose, the major component of FDA-approved agar, gained particular attention for the fabrication of advanced delivery systems, as sophisticated carriers for therapeutic agents [87], and these have been tested in human trials [88]. Alginate is also FDA approved and often used in drug- and protein-delivery systems and for wound healing treatments [89]. Cellulose is one of the most abundant biodegradable materials in nature, and has been widely used in medical applications such as wound dressing [90], tissue engineering [91], controllable drug delivery system [92], treatments [93,94], and as a excipient in the pharmaceutical industry. Poly(ethylene glycol) (PEG), poly(ε-caprolactone) (PCL), and poly(lactide-co-glycolide) (PLGA) are synthetic polymers that have been investigated in clinical trials and approved by the FDA for safe use in humans. PEG has been widely utilized in biomedical applications, such as bioconjugation, in which it is either directly conjugated with drugs or attached to the surface of drug-encapsulating nanomaterials (a technique known as PEGylation) [95], in drug delivery [96], in biosensing (e.g., for electrochemical, optical and mass-based biosensors) [97], and in tissue engineering [98]. PCL has recently been considered for biomedical applications, including bone tissue engineering [99], drug delivery [100], and as dermal and subdermal fillers, since it is an excellent collagen stimulator [101]. The other attractive polymer for the fabrication of drug delivery and tissue engineering applications is PLGA [102]. There are many commercially available PLGA-based medical products, including membranes, sponges, powders, gels, and sutures [103], as well as scaffolds, films, and micro- and nanoparticles utilized for the dental field [104].

The main barriers for clinical translations are associated with the use of non-FDA approved, new chemical entities. Indeed, whilst many of the non-functionalized polymers mentioned above are already FDA-approved, additional and separate approval is required after functionalization and for each novel biomaterial-based medical device. Obtaining such approval typically requires a lengthy and costly regulatory trajectory, but is a strict necessity before the materials can be tested on humans and ultimately become part of clinical practice.

Consequently, and given that the field of biodegradable and biocompatible optical fibers is still in its infancy, it is difficult to pinpoint a “killer application” for such devices today. At the same time, owing to the progress made so far, it is our belief that they hold promise for becoming enabling tools for diverse medical applications, such as deep-tissue light delivery, PDT, imaging, and sensing. PDT is clinically approved for treating a number of cancers (bladder, lung, skin, esophageal, brain, and ovarian), and has also been demonstrated by means of optical fibers [105]. Commercially available polymer or silica based optical fibers (e.g., manufactured by Medlight, Lausanne, Switzerland [106]) that can be used for that purpose have a partially stripped cladding, and their core is coated with a light-scattering material along the desired length of the diffusing tip. Considering that PDT is already applied in the clinic, one can conjecture that material solutions supporting this treatment could go to market first.

Moreover, there is indeed an immense potential for exploring the possibilities of applications in which these optical fibers could be employed in the near future, such as light-induced manipulation of cells by means of optogenetics, continuous in vivo monitoring, label-free optical sensing (e.g., by means of Bragg grating peak wavelengths shifts), and light triggered drug release implanted in the microstructures of such fibers. Optical fibers may become frequently used parts of implantable optical components within fully bioresorbable medical devices [107], such as the reported biosensors [108], i.e., millimeter-scale bioresorbable Fabry-Pérot interferometers that enabled continuous measurements of pressure and temperature [109], or in a device used for the continuous monitoring of cerebral temperature, oxygenation, and neural activity for the spectroscopic characterization of targeted tissues and biofluids in living mice [110], and other devices for nerve regeneration and optogenetics [111].

The capability to combine modern nanotechnologies with optical fibers is also an emerging trend in advanced biosensing technology. Biological detection and optical manipulation of biospecimens such as organic nanoparticles, biomolecules, and bacterial viruses, for example, is being investigated by means of conventional silica based optical fibers [112]. Micro- and nano-optical fibers, including tapered fibers, plasmonic fibers, microlens-based fibers, and nanowires, are excellent candidates because of their low optical loss, tight optical confinement, and large fractional evanescent fields, making them a novel miniaturized platform for optical sensing with higher sensitivity and spatial resolution [113]. These fibers could be fabricated from biocompatible and bioresorbable materials to guarantee a minimally invasive procedure and limit cytotoxicity.

The analysis of the current state-of-the-art revealed a number of important findings on various biocompatible specialty optical fibers since the first report on bioresorbable cellulose-based fiber was presented in 2007. Remarkably, biocompatibility, degradation, and optical performance have been successfully shown in vitro and in animal models for some fibers, whilst others have been employed for several proof-of-concept demonstrations. In spite of these major achievements, several supplemental challenges should still be addressed before large-scale practical applications in both preclinical and clinical scenarios can be envisaged. These include, for example, the development of efficient connectors for terminating specialty optical fibers and connecting them to, or disconnecting them from, standard lead optical fibers that remain outside a patient’s body.

We are convinced that the unique properties of light guiding biomaterial-based fibers can support and improve medical diagnosis and therapy and enable new future applications in photomedicine. The potential of bioresorbable and biocompatible optical fiber technology is immense, and therefore we expect research on this topic to intensify in the coming years.

## Figures and Tables

**Table 1:** Summary of biomaterials used for optical fibers.

Material Type	Material Example	Advantages	Disadvantages
Natural	Proteins: silk Polysaccharides: alginate, cellulose, agarose	biocompatibility and biodegradability	poor designability, typically limited sources and amount, variability from batch-to-batch, low mechanical strength, can be immunogenic
Synthetic	Hydrogels: PEG, Pluronic Citrate-based elastomers: poly(octamethylene citrate) (POC), poly(octamethylene maleate citrate) (POMC), Polyesters: poly(L-lactic acid) (PLLA), poly(D,L-lactic acid) (PDLLA), poly(L-lactic-co-glycolic acid) (PLGA), poly(D,L-lactic-co-glycolic acid) (PDLGA), poly-ε-caprolactone (PCL) Inorganic materials: calcium-phosphate glass (PGs)	flexible design, controllable biodegradability, adjustable physical, mechanical and chemical properties	biocompatibility should be verified and confirmed, rigidness and brittleness for glass

**Table 2:** Summary of biodegradable and biocompatible optical fibers and waveguides manufactured by means of thermal drawing from preforms.

Material Type	Preform Fabrication	Fiber Type	Optical Loss	Reference
CB, HPC	CB commercial tubes filled with HPC powder	Double core porous, diameter: 450 µm	1.1 dB/cm at 630 nm	[51]
CA, PLLA	Co-rolling CA commercial film with PLLA cast film	Core-cladding, SI diameter: 860 µm	9.8 dB/cm at 633 nm	[52]
CB	CB commercial tubes	Hollow core diameter: 393 µm	2.2 dB/cm at 633 nm	[52]
CB, PCL	CB commercial tubes filled with PCL powder	Core-cladding, SI diameter: 420 µm	6.7 dB/cm at 633 nm	[52]
CB, PCL	Inserting small diameter CB tube into a larger diameter CB tube and filling the space between with PCL powder	Multiple core, SI diameter: 410 µm	8.3 dB/cm at 633 nm	[52]
CB, HPC	Inserting small diameter CB tube into a larger diameter CB tube and filling the space between tubes with HPC powder dissolved in water	Porous cladding, SI diameter: 415 µm	3.1 dB/cm at 633 nm	[52]
PLLA PLGA	No preform. Simplified thermal drawing directly from molten powder using glass capillary tubes	Unclad PLLA fibers, Unclad PLGA fibers, diameter: 220 µm	1.6 dB/cm at 473 nm	[54]
PDLLA	Melting the PDLLA granulates in form of a homogenous rod	Unclad PDLLA fibers diameter: 600, 300, 200 µm	0.11 dB/cm at 772 nm 0.17 dB/cm at 636 nm	[55]
PDLLA, PDLGA	Rod-in tube technique: melting the PDLLA granulates in Teflon^®^ molds in the form of a tube and PDLGA granulates in form of homogenous	Core-cladding, SI fibers, diameter: 1000 ± 50 µm, where core is 570 ± 30 µm	0.26 dB/cm at 950 nm 0.50 dB/cm at 633 nm	[56]
PMMA, PLGA, PLGA75 PLGA85	Rectangular, cylindrical, and multimaterial preforms prepared by hot-pressing a base polymer plate (i.e., PMMA or PLGA) and milling channels along the preforms to be filled with PLGA	Rectangular fibers with channels, Unclad cylindrical fibers	-----	[57]
PCL, PLGA85	PLGA tube fabricated in a hollow core mold in the oven. The mold was taken out and the core was filled with PCL powder, and heated	Core-cladding fibers	-----	[57]
PCL	PCL preforms prepared by melting PCL pellets inside polypropylene and Teflon molds of circular, three- and four-leaf cross-sectional shapes at 80 °C for 17 h	Unclad solid-core and grooved fibers; diameter around 700 μm, hollow core with internal diameter 200 μm	1.5 dB/cm at 635 nm in PBS and 2.5 dB/cm over 21 days of immersion in PBS	[58]
PGs	Rod-in-tube technique: rod made from a previously drawn thicker rod, and tube made by rotational casting	Core-cladding, SI, MMF Core-cladding, SI, SMF inscribed with FBG	0.019 dB/cm at 1300 nm 0.047 dB/cm at 633 nm	[41,59]
PGs	Direct extrusion of outer tube and standard stack-and-draw technique by assembling extruded capillaries within the tube	Microstructured fibers	-----	[61]

**Table 3:** Summary of optical fibers and waveguides manufactured by means of extrusion, compression molding, and solution casting followed by curing.

Material Type	Fabrication Technique	Fiber Type	Optical Loss	Reference
Silk fibroin	direct ink extrusion applied under pressure from aqueous silk solution	unclad silk fibers on glass slides diameter: 5 μm	0.25 dB/cm at 633 nm	[67]
Spider silk	native spider silk directly woven by spiders	unclad silk fiber diameter: 5.6 μm	10 dB/cm at VIS	[68]
PEGDA(DTT) as core (Pluronic F127-DA) as cladding	extrusion printing technique using a commercial 3D-bioscaffolder, followed by UV photopolymerization	unclad hydrogel-based fibers and core-cladding hydrogel-based fiber diameter: core from 340 to 640 µm and total diameter of 1.02 mm	0.1 dB/cm at 520 nm and 0.4 dB/cm at 405 nm	[69]
Regenerated cellulose as core Cellulose acetate as cladding	the regenerated cellulose core was produced from (EMI- M) OAc by using dry-jet wet spinning in water bath as a coagulant. The cladding was produced by coating the cellulose core with cellulose acetate dissolved in acetone.	core-cladding, SI diameter: core: 210 µm, cladding: 3.40 µm	6.3 dB/cm at 1300 nm, ~ 10 dB/cm in the 750–1350 nm	[70]
PLA and PLGA	compression molding of polyester powders and laser cut of polymer sheets	planar waveguide	1.6 dB/cm at 635 nm	[76]
Silk fibroin and silk hydrogel ^1^	silk solution was cast into a mold as a core and dip-coating of core in silk hydrogel solution prior to gelation in a Teflon tube as a cladding	core-cladding diameter: 3 mm	2 dB/cm at 540 nm	[73]
Recombinant spider silk protein and regenerative silkworm silk protein	genetically engineered spider silk protein generated by means of biosynthesis. Recombinant spider silk protein was dissolved in hexafluoro-2-propanol at 37 °C overnight, whilst regenerated silkworm silk solution was directly cast into Teflon^®^ tubes with inner diameters of 800 µm. Protein solutions in the molds were heated at 60 °C for 7 days for complete solidification.	unclad fibers diameter: 700 µm	0.8 dB/cm at 635 nm	[74]
PEG as core and alginate as cladding	precursor solution for PEG hydrogel was injected into mold tube and photo-crosslinked by UV. The PEG core was dipped multiple times in a sodium alginate and calcium chloride to form cladding	core-cladding, SI, diameter: core: 250–800 µm, cladding 100–150 µm	0.42 dB/cm at 492 nm	[75]
PEG	photopolymerization of the precursor solution of PEG by UV	planar waveguides	----	[76]
Ca^2+^ with Na alginate polyacrylamide (PAAm) hydrogel	precursor solution of acrylamide with Na alginate was injected into a silicone tube mold using a syringe and crosslinked at 50 °C under UV for 30 min.	unclad fibers, diameter	0.56 dB/cm at 532 nm	[77]
Ca^2+^ with Na alginate polyacrylamide (PAAm) hydrogel	precursor solution of acrylamide with Na alginate was injected into a silicone tube mold using a syringe and crosslinked at 50 °C under UV for 30 min. The unclad fiber was dipped in an Na-alginate-polyacrylamide precursor. The clad-coated core fiber cured by UV irradiation for 30 min. Fiber was immersed in an aqueous solution of CaCl_2_ for ionic cross-linking of alginate by Ca^2+^ for robustness	core-cladding, SI diameter: 750 μm core and 1100 μm cladding	0.45 dB/cm at 532 nm	[77]
POC pre-polymer, citric acid (CA) and 1,8-octanediol (OD) as cladding: POMC pre-polymer, CA, maleic anhydrate (MAn) and OD as core	thermal crosslinking of a pre-polymer cladding material in the form of a tube surrounding metal wire. Air pressure infiltration of liquid POMC into the pre-polymerized POC tube, which followed the thermal crosslinking of both at 70 °C for 7 days.	core-cladding, SI, diameter: 750 μm	0.4 dB/cm at 633 nm	[78]
Agarose	boiled agar solution poured into the glass mold tube with rods, cooled down and released after solidification	structured fiber with 6 holes diameters of core: 0.64 mm, cladding: 2.5 mm, and air holes: 0.5 mm	3.23 dB/cm at 633 nm	[79]

^1^ Hydrogel prepared by mixing silk fibroin solution, horseradish peroxidase (10 U/mL), and 10 μL/mL of 1% hydrogen peroxide.
